# Supplementary material for: Co-Expression Networks for Causal Gene Identification Based on RNA-Seq Data of Corynebacterium pseudotuberculosis
Source: Genes (Basel). 2020 Jul 14;11(7):794. doi: 10.3390/genes11070794 (PMC7397307; doi:10.3390/genes11070794)
Supplement: Supplementary file 1 [file genes-11-00794-s001.zip › Supplementary materials/Files 2-Supplementary_Figures.pdf]

Supplementary Materials: Co-expression networks for causal gene identification based RNA-seq data of *Corynebacterium pseudotuberculosis*

Edian F. Franco [1,4], Pratip Rana [2], Ana Lidia Queiroz Cavalcante [1], Artur Silva [1], Adriana R. Carneiro Folador[1], Vasco Azevedo [3], Preetam Ghosh [2‡] and Rommel T. J. Ramos [1,3‡] *

1. Supplementary Figures

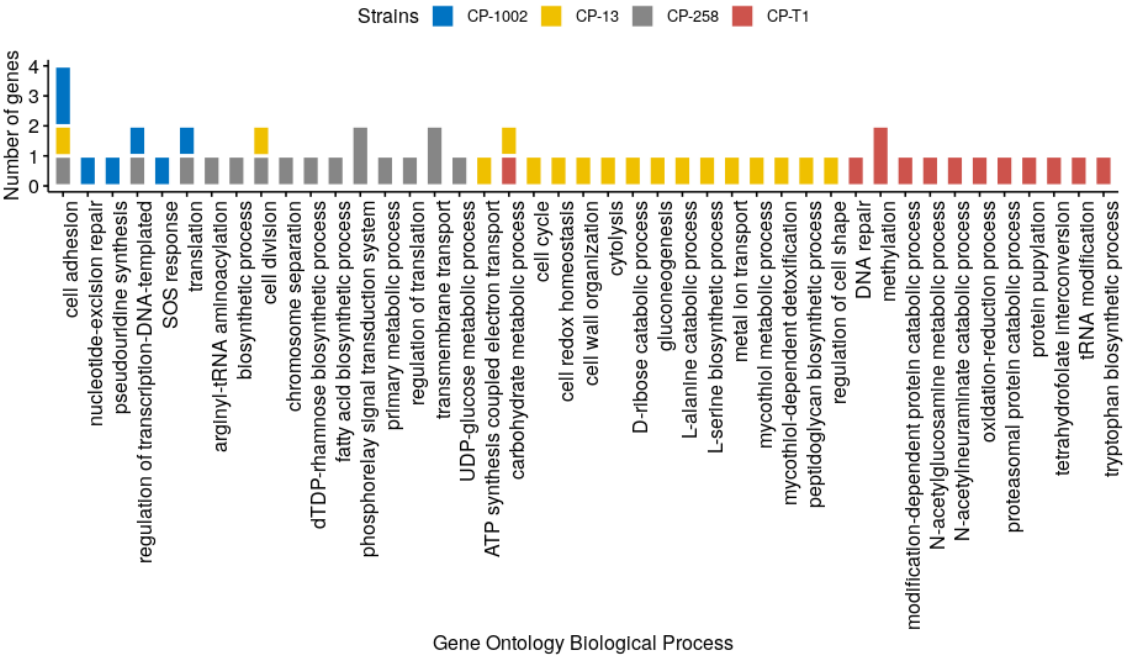

Figure S1. Biological process results of whole expressed genes in CP-1002, CP-258, CP-13, CP-T1

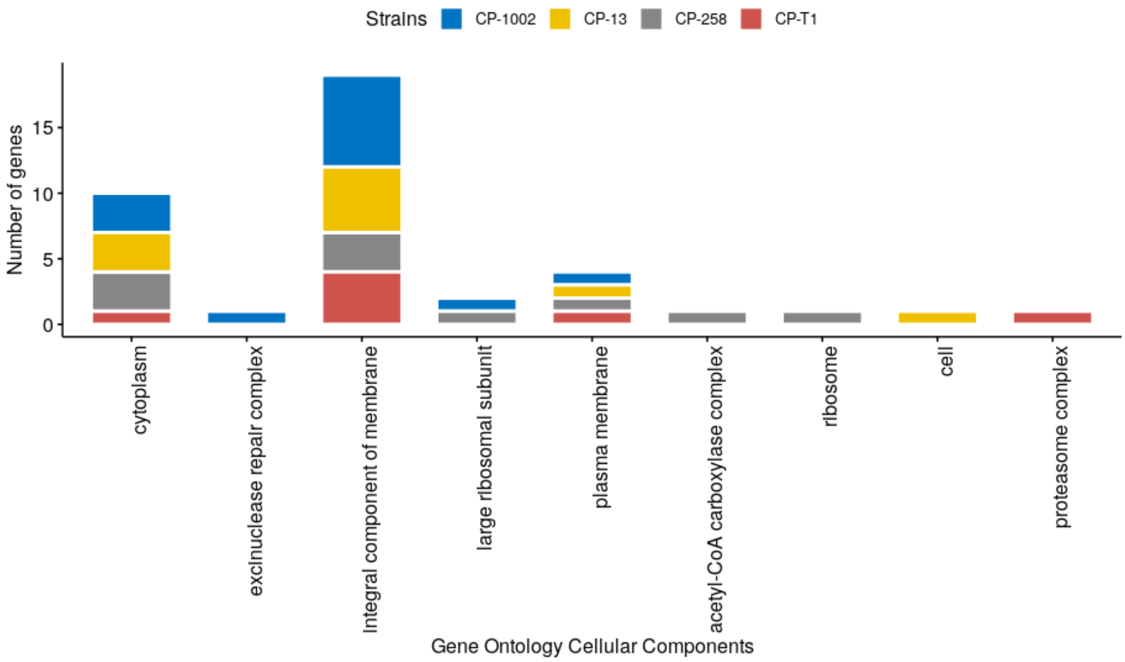

Figure S2. Cellular Components results of whole expressed genes in CP-1002, CP-258, CP-13, CP-T1

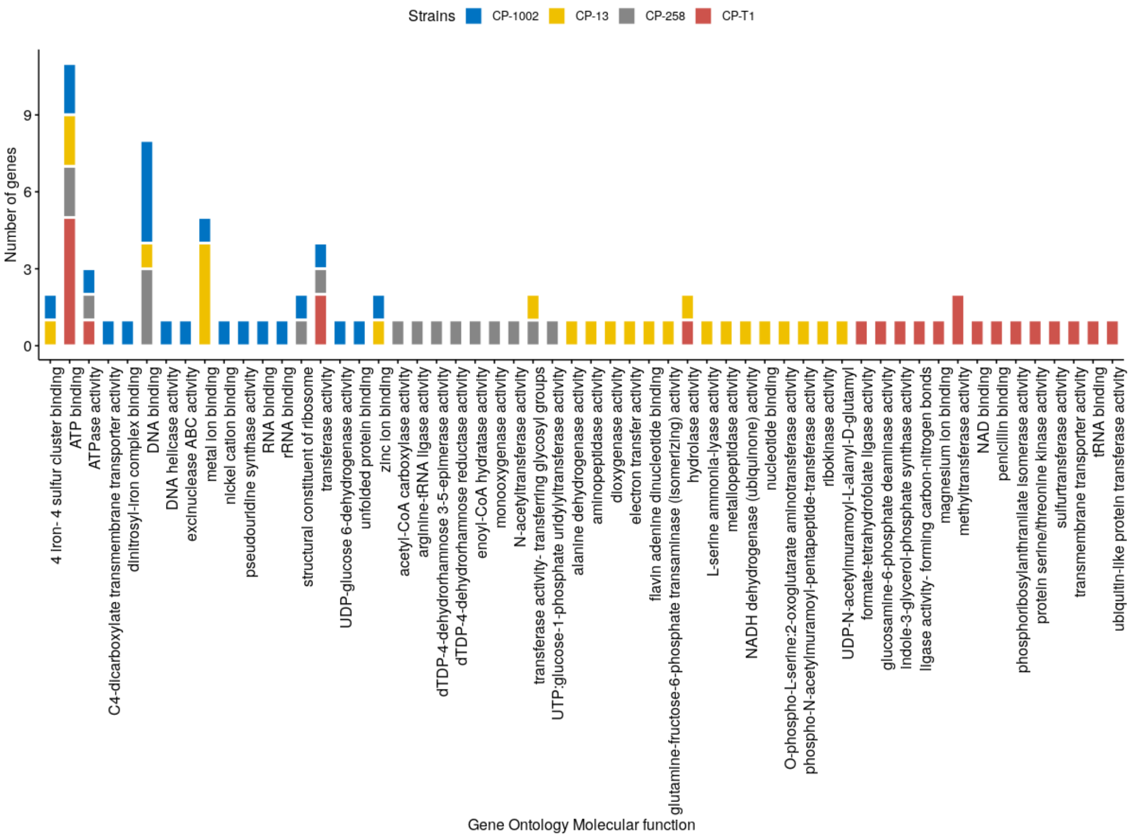

Figure S3. Molecular function results of whole expressed genes in CP-1002, CP-258, CP-13, CP-T1

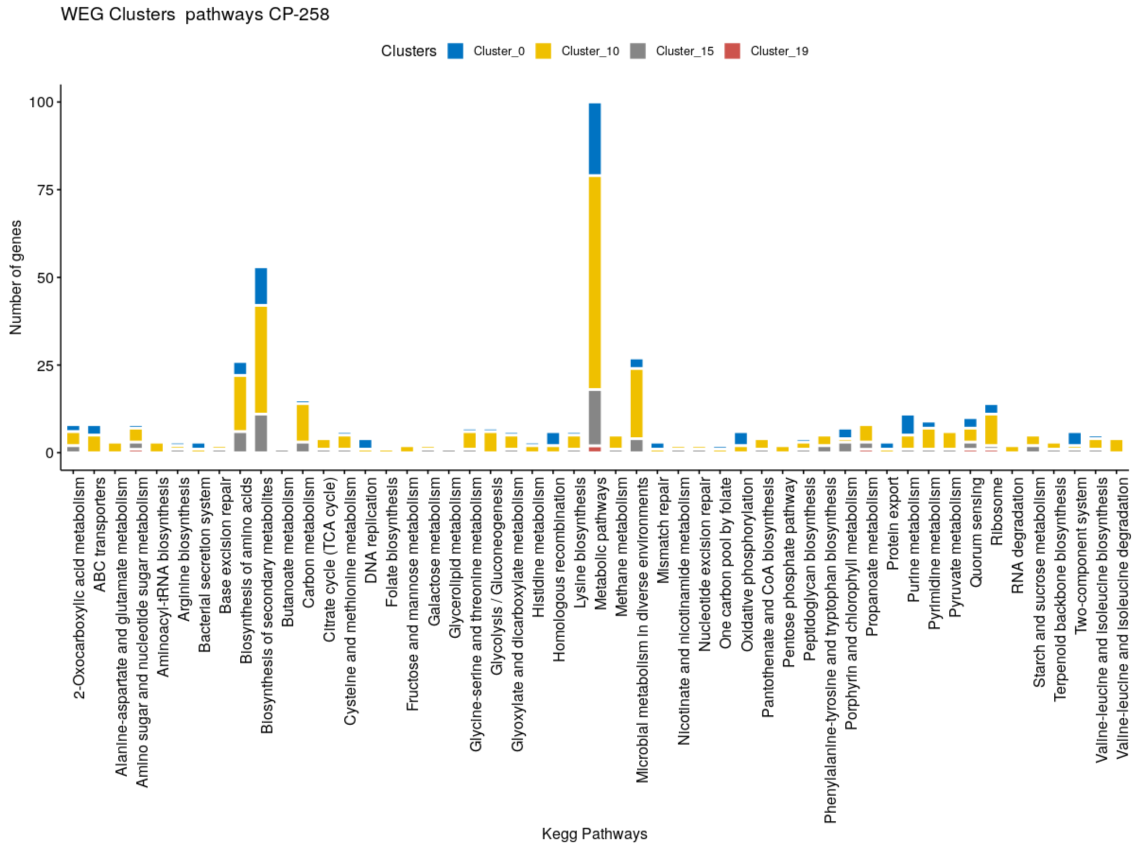

Figure S4. Pathways of the clusters where the influential genes are present in the whole expressed genes network in CP-258.

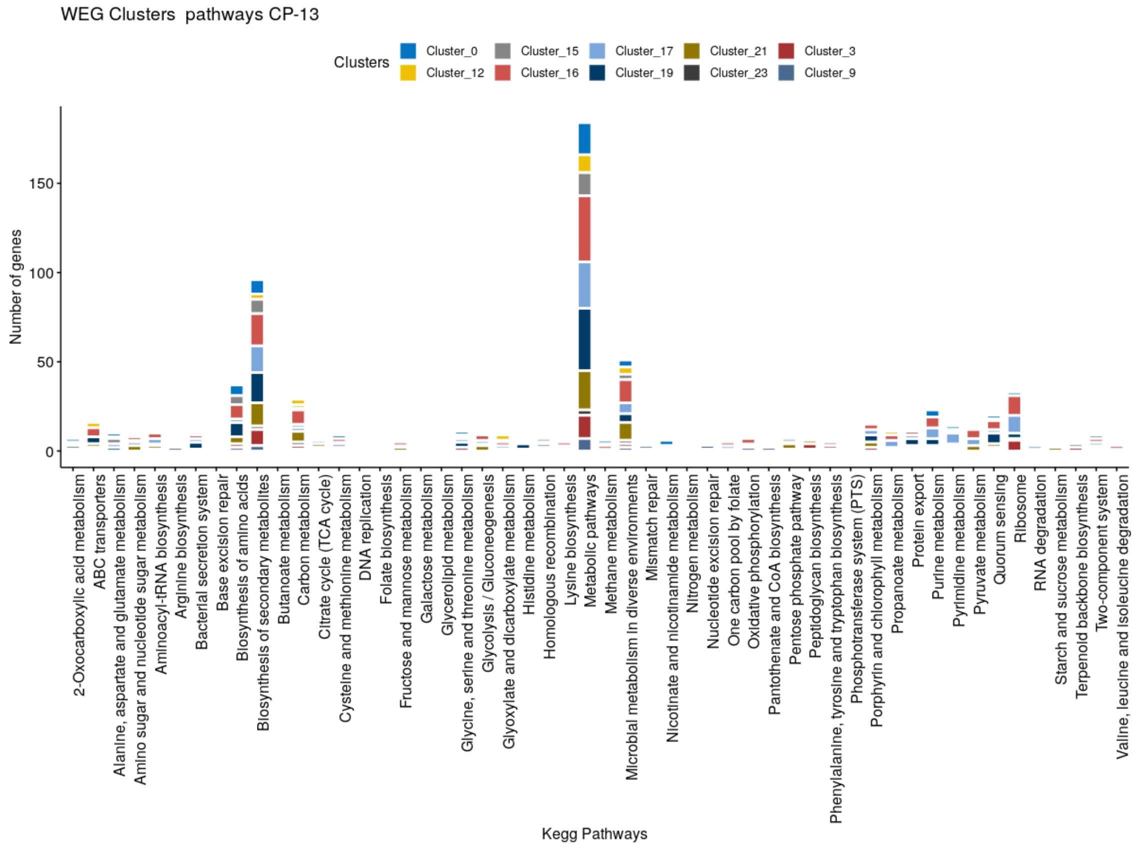

Figure S5. Pathways of the clusters where the influential genes are present in the whole expressed genes network in CP-13.

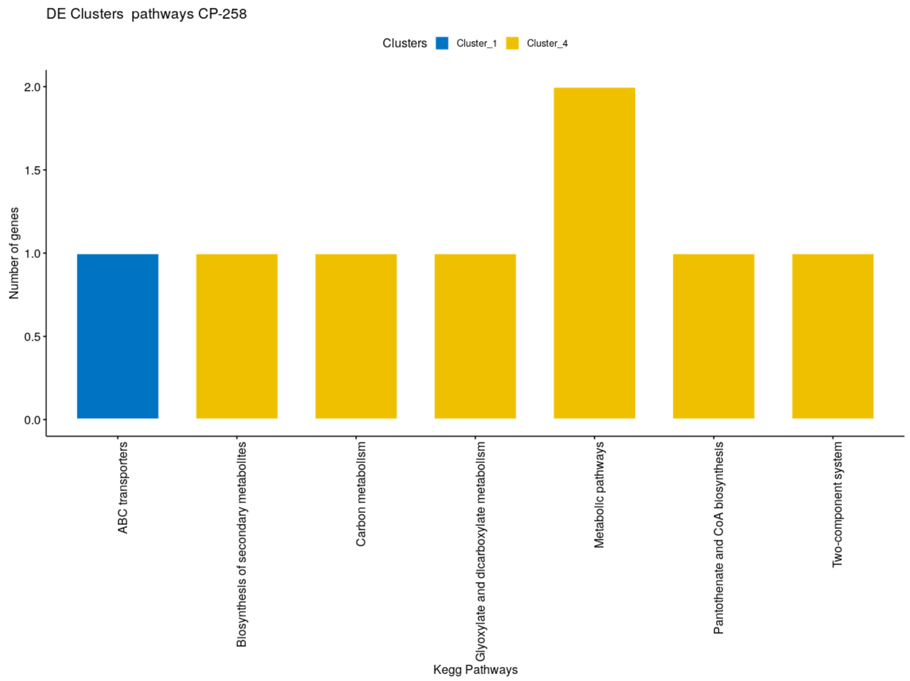

Figure S6. Pathways of the clusters where the influential genes are present in the Deferentially expressed genes network in CP-258.

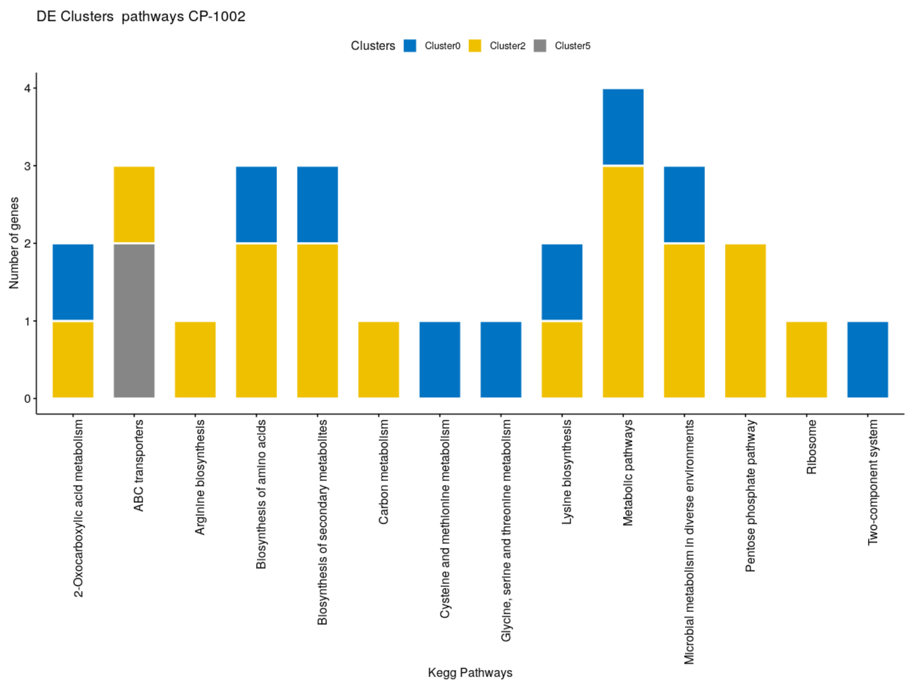

Figure S7. Pathways of the clusters where the influential genes are present in the Deferentially expressed genes network in CP-1002.

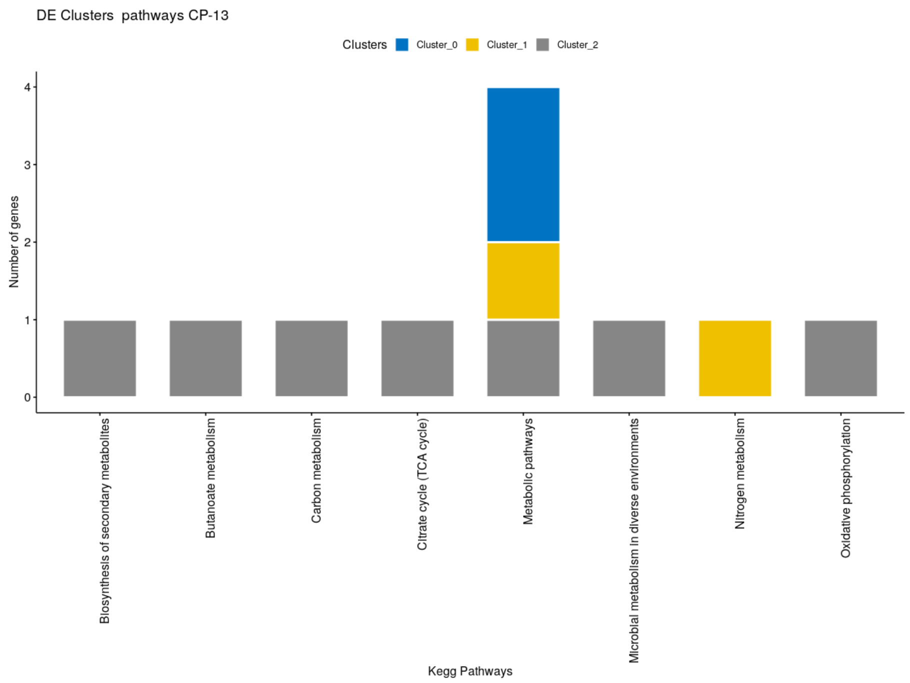

Figure S8. Pathways of the clusters where the influential genes are present in the Deferentially expressed genes network in CP-13.

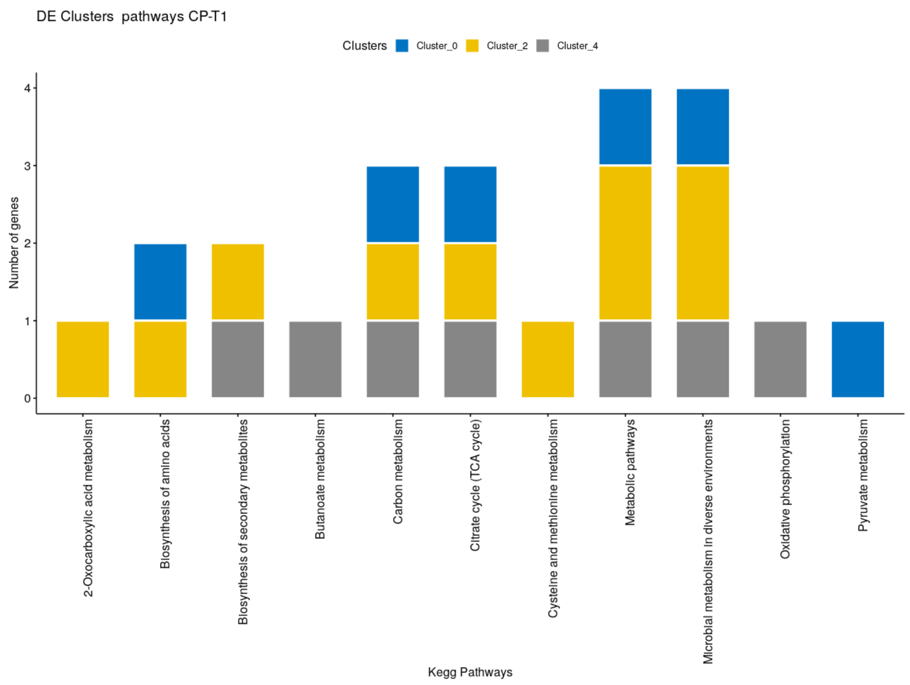

Figure S9. Pathways of the clusters where the influential genes are present in the Deferentially expressed genes network in CP-T1.
